# 

**Published:** 1843-04

**Authors:** 


### CONTENTS

OF NO. IV.

---

### ORIGINAL COMMUNICATIONS.

### ESSAYS AND CASES.

Art. I.—Post-mortem Researches. By Bennet Dowler, M. D., of New Orleans. - - - - - 241

Art. II.—A Therapeutical arrangement of the Materia Medica; or the Materia Medica arranged upon Physiological Principles, and in the order of the general practical value which remedial agents hold under their several denominations, and in conformity with the Physiological doctrines set forth in the Medical and Physiological Commentaries. By Martyn Paine, M. D., A. M., &c. - - - - - - - 237

Art. III.—The Diseases of Females: including those of Pregnancy and Childbed.—By Fleetwood Churchill, M. D., author of the "Theory and Practice of Midwifery." Second American edition. - - 281

Art. IV.—A System of Anatomy for the use of Students of Medicine. By Caspar Wistar, M. D., late Professor of Anatomy in the University of Pennsylvania. 282

SELECTIONS FROM AMERICAN AND FOREIGN JOURNALS.

On a Variety of False Aneurism. - - - 283
A Submarine Thermometer, - - - - 285
The Salts of Quinia. - - - - - 286
Consequences of the Accidental Introduction of pieces of Glass into the Body. - - - - 286
Report of a case of Diseased Ear, which produced death in a child of four years of age. - - 288
Encephalo-Spinal Meningitis. - - - - 290
Galvanic Forceps. - - - - - 292
Nursery Treatment of Infants, submitted to Prince Albert. 293
New Process for Anatomical Injections. - - 295
Syphilitic Retraction of the Muscles. - - - 296
Prevention of Lead Colic. - - - - 297
Robertson on the Cause of Caries of the Teeth. - 297
Iodide of Potassium in Atonic Ulcers. - - - 299
Medical Topography of La Plata. - - - 299
The Chigoe, or Gigger. - - - - - 301
On Contusions of Muscles. - - - - 302
A Population getting Shorter. - - - - 306
Case of Abcess in the Walls of the Uterus, communicating with the Rectum. - - - - - 307
Tuberculous Disease of the Spinal Marrow. - - 310
Latent Insanity. - - - - - 311
Public Speaking. - - - - - 312

ORIGINAL INTELLIGENCE.

Travelling Editorials, - - - - - 313
Medical Convention of Ohio. - - - - 315
Artificial Anus. - - - - - 316
Louisville Medical Institute. - - - - 316
Meum and Teum. - - - - - 317
Quadrupeds of North America. - - - - 318
Anonymous Pamphleteers. - - - - 319
Medical Resignation. - - - - - 320
New York Lancet. - - . - - 320

